# Comparative Genomics: Insights on the Pathogenicity and Lifestyle of *Rhizoctonia solani*

**DOI:** 10.3390/ijms22042183

**Published:** 2021-02-22

**Authors:** Nurhani Mat Razali, Siti Norvahida Hisham, Ilakiya Sharanee Kumar, Rohit Nandan Shukla, Melvin Lee, Mohd Faizal Abu Bakar, Kalaivani Nadarajah

**Affiliations:** 1 Department of Biological Sciences and Biotechnology, Faculty of Science and Technology, Universiti Kebangsaan Malaysia, Bangi 43600, Selangor, Malaysia; hanirazali93@gmail.com (N.M.R.); vahidadesham@gmail.com (S.N.H.); ilakiya95sharanee@gmail.com (I.S.K.); 2 Bionivid Technology Pte Ltd., 209, 4th Cross Rd, B Channasandra, East of NGEF Layout, Kasturi Nagar, Bengaluru 560043, Karnataka, India; rohit@bionivid.com; 3 Codon Genomics Sdn. Bhd., No 26, Jalan Dutamas 7 Taman Dutamas Balakong, Seri Kembangan 43200, Selangor, Malaysia; melvinlee@codongenomics.com; 4 Malaysia Genome Institute, Kajang 43000, Selangor, Malaysia; mfaizal@mgi-nibm.my

**Keywords:** *Rhizoctonia solani*, anastomosis group (AG), comparative genomics, Genome Annotation, repeat element, CAZy, pathogenicity genes, transposable element, single copy orthologs, synteny

## Abstract

Proper management of agricultural disease is important to ensure sustainable food security. Staple food crops like rice, wheat, cereals, and other cash crops hold great export value for countries. Ensuring proper supply is critical; hence any biotic or abiotic factors contributing to the shortfall in yield of these crops should be alleviated. *Rhizoctonia solani* is a major biotic factor that results in yield losses in many agriculturally important crops. This paper focuses on genome informatics of our Malaysian Draft *R. solani* AG1-IA, and the comparative genomics (inter- and intra- AG) with four AGs including China AG1-IA (AG1-IA_KB317705.1), AG1-IB, AG3, and AG8. The genomic content of repeat elements, transposable elements (TEs), syntenic genomic blocks, functions of protein-coding genes as well as core orthologous genic information that underlies *R. solani*’s pathogenicity strategy were investigated. Our analyses show that all studied AGs have low content and varying profiles of TEs. All AGs were dominant for Class I TE, much like other basidiomycete pathogens. All AGs demonstrate dominance in Glycoside Hydrolase protein-coding gene assignments suggesting its importance in infiltration and infection of host. Our profiling also provides a basis for further investigation on lack of correlation observed between number of pathogenicity and enzyme-related genes with host range. Despite being grouped within the same AG with China AG1-IA, our Draft AG1-IA exhibits differences in terms of protein-coding gene proportions and classifications. This implies that strains from similar AG do not necessarily have to retain similar proportions and classification of TE but must have the necessary arsenal to enable successful infiltration and colonization of host. In a larger perspective, all the studied AGs essentially share core genes that are generally involved in adhesion, penetration, and host colonization. However, the different infiltration strategies will depend on the level of host resilience where this is clearly exhibited by the gene sets encoded for the process of infiltration, infection, and protection from host.

## 1. Introduction

*Rhizoctonia solani* is a soilborne pathogen that is capable of causing seedling damping-off, sheath blight, root rot, collar rot, stem canker, crown rot, bud and fruit rots, and foliage blight on a variety of susceptible agriculturally important crops like soybean, cotton, canola, wheat, beet, potato, rosemary, and turfgrass species [1]. *R. solani* is divided into 14 anastomosis groups (AG) based on the hyphal anastomosis reaction (AG1-AG13) [2], which is further refined to subgroupings such as AG1-IA, AG1-IB, and AG1-1C. Strains classified within the same AG have a close relationship to each other and most likely have the same host range [3]. Currently, disease management of *R. solani* relies on fungicide application, which can be detrimental to the environment. Besides the application of fungicide, research has been conducted in breeding for resistance. While several QTLs [4,5,6,7,8] have been identified from previous studies for rice sheath blight, most only contribute towards partial resistance with no identified major resistance genes [9,10,11,12]. The constant breakdown of resistance and partial tolerance of resistance to *R. solani* as seen in most crops has prompted the need to decipher what is the root cause driving the virulence of this organism through molecular dissection.

*R. solani* infects plant host via specialized infection hyphae or infection structures, namely infection cushions and/or lobate appressoria. The penetration of the cuticle is achieved through turgor pressure and enzymatic degradation of host tissue [2]. In *R. solani*, pathogenesis is brought about by secreted compounds like phytotoxins, cell wall degrading enzymes, and other extracellular enzymes that act as pathogenicity determinants for the host [13,14]. It is believed that these compounds are secreted before or during pathogen colonization starting from infiltration to propagation [15]. Through the progression of the disease infiltration, the necrotic lesions spread and result in the death and decay of host tissue. Verwaaijen et al. (2017) hypothesized that in the early phase of interaction of fungi with the host, genes that suppress host defense will be expressed and secreted first before phytotoxins [16]. Some genes activated in *R. solani* are cell wall degrading enzymes (CWDE) such as cellobiose dehydrogenase, β-xylanase, β-glucanase, β-glucosidase, cutinase, and glucosyl transferase that play an important role in necrotrophy. Chitin deacetylase encoding gene though activated during necrotrophy was also implicated in pathogen protection from CWDE. In addition to CWDE, sugar transporter genes, hexose transporters, and effectors were upregulated in the initial establishment of infection and progressed into the necrotrophic phase [17]. Once these genes are expressed, it is expected that phytotoxins [3-methylthiopropionic acid, 3-methylthioacrylic acid] are then secreted to complete the infection arsenal.

In this study, four different AGs were used in a comparative analysis with our Draft AG1-IA 1802/UKM (here after referred to as Draft AG1-IA). AG1-IA, AG1-IB, AG3, and AG8 exhibit host specificity that is from narrow to broad. AG1-IA has been associated with the development of rice sheath blight and foliar blight in other graminaceae such as soybean [18,19]. The ability to infect mostly graminaceae has classified this AG into a narrow, monocot specific host range group [19]. However the similarity of disease symptoms makes it difficult to distinguish the various *Rhizoctonia* species as some AGs like AG1-IA fuse well with isolates of IB and IC [20]. AG1-IB, a subgroup of AG1, is classified as intermediate to broad host range where it has been reported to infect monocots and dicots. While being a major pathogen of lettuce and cabbage it also infects some common host of AG1-IA. AG1-IB is the causative agent of web blight and bottom rot [16]. Next we have AG3, which is classified as narrow to intermediate host range fungi. AG3 predominantly infects potatoes and has been listed to cause stem canker, black scurf, leaf blight, and brown spot in plants [21]. Finally there is AG8, which is a broad host pathogen that causes bare patches in cereals and other crops [22,23].

Field isolates of *R. solani* have shown an increased variability in pathogen complexity making it difficult for researchers to select for resistant host genotypes and deploy tolerant varieties [24]. Therefore, knowing the variability between AGs will be helpful for researchers to breed for disease resistance. Knowing the variability in the virulence and pathogenicity patterns of this pathogen also helps in the evaluation and identification of resistant and susceptible genotypes [25]. Over the past decades, efforts on sequencing and analyzing the genome of *R. solani* isolates has presented opportunities for us to tease apart the genetic determinants underlying pathogenicity and host specificity [3,26]

Previous studies have suggested that transposable elements (TE) are one of the possible genetic determinants that drive genome plasticity, genome expansion, promotion of pathogenicity in fungi through mutations, and the cause behind chromosomal rearrangement [27,28,29,30,31,32]. However, the potential role of TEs in *R. solani* lifestyle and evolution is poorly investigated as compared to other pathogens. The differences underlying the pathogenicity mechanism, host specificities, lifestyle, and evolution between these different anastomosis groups can be unveiled by investigating the genome informatics of these AGs.

Therefore, in this paper, we endeavor to understand the specifics behind the pathogenic lifestyle of *R. solani* through comparative genome analyses among five different AGs (Draft AG1-IA, China AG1-IA, AG1- IB, AG3, and AG8), while exploring the potential role of TE in pathogenicity, an area that has not been comprehensively discussed previously. Further, similarities or differences in the genome profiles between Draft AG1-IA and the China isolate (both of the same anastomosis groups) will also reveal geographical effect on strain variation, genome variability, and their pathogenicity strategy. Through the predictive influence of TE on pathogenicity and the identification of the core arsenal of these five isolates, we hope to predict a pathogenic lifestyle for this fungus and use the fundamental knowledge derived to development an efficacious *R. solani* disease management strategies in the future.

## 2. Results and Discussion

### 2.1. Genome Annotation of Draft AG1-IA & Comparative Genome Assembly Statistics

Our draft genome assembly of *R. solani* AG1-IA (Malaysian isolate) was published under the accession GCA_001899475.2 [33]. Between the specified 14 AG’s of *R. solani*, there are 6 genome establishments (including our Draft AG1-IA) with their comparative genome informatics shown in Table 1.

Gene prediction was accomplished by Gene-Mark-ES fungal version, an ab initio gene predictor program followed by a manual curation, which detected 10,037 protein coding genes, which is slightly lower compared to China AG1-IA (with 10,489 protein coding genes) by 4.31%. The comparison with other *R. solani* genomes is provided in Table 1.

A total of 7491 genes, which accounts for 75% of the total genes, were annotated via the fungal database in NCBI. Predicted genes were also subjected to gene characterization through ontologies and domains assignment. Most of these genes mapped to molecular functions that were predominantly ATP binding, zinc ion binding, and those with hydrolase activity. Meanwhile carbohydrate metabolic processes, transmembrane transport, and metabolic processes were the most dominant ontologies in biological function, while integral components of membrane, nucleus and cytoplasm are the top three cellular components. Further, InterPro annotations demonstrated WD40 repeat, P-loop containing nucleoside triphosphate hydrolase, and Cytochrome p450 as the most abundant domains [34]. These dominant ontologies, domains, and integral components may be linked to the postulated necrotrophic action of *R. solani* and will be discussed further under the Section on Single Copy Orthologs.

### 2.2. Repeat Element Identification and Characterization of Transposable Element

**Figure 1:**
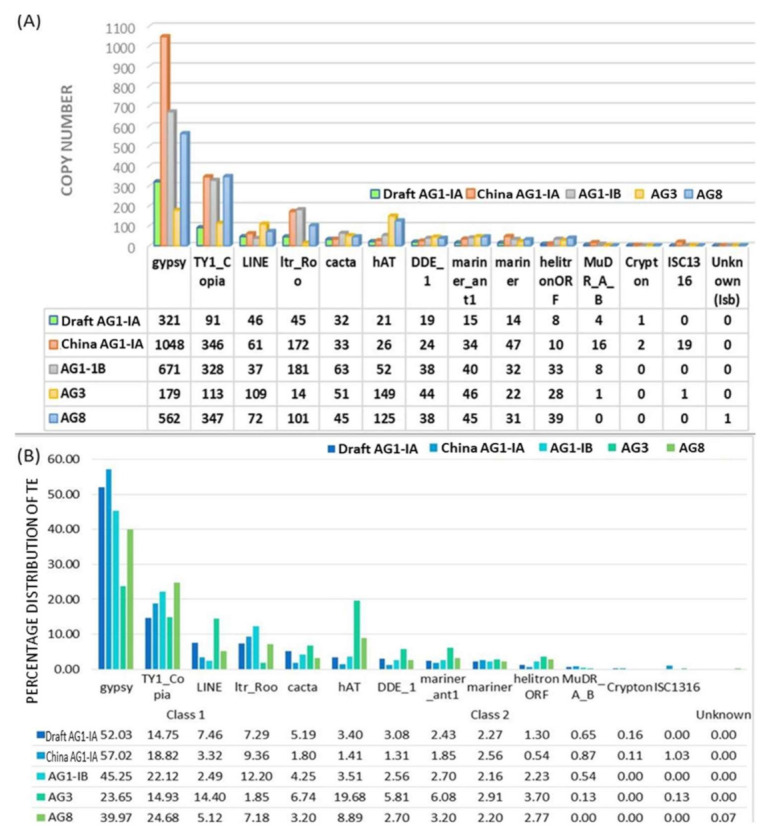
Transposable elements (TE) landscape and statistics. (**A**) Distribution of TE copy number in Class I, and II according to superfamilies in five AGs of *R. solani*. (**B**) Percentage distribution of TE in Class I, and II according to superfamilies in all five AGs of *R. solani*.

Repeat elements distribution was compared between Draft AG1-IA, China AG1-IA, and AG8 as these publications provided extensive informatics on the breakdown of repeat elements. For Draft AG1-IA, repeat elements were identified by using a combinatorial approach of de novo and homology-model based analysis, which is similar to a certain degree to the tools used for the other two genomes (Table 2). A comparison between the tools used and the percentage of repeat elements identified is presented in Table 2. The results showed that the Draft AG1-IA is made of 4.86% of repeat elements, which is slightly lower than China AG1-IA (5.27%), and is significantly different from the higher values exhibited in AG8 (10.03%). This is attributed to the high portion of unknown repeats (4.17%) and unspecified repeats (1.03%) in AG8 in comparison to the Draft AG1-IA [unknown repeats (1.26%) and unspecified repeats (1.19%)] and AG1-IA (0.007%). Draft AG1-IA consists of 1.07% Class 1 TEs and 0.28% Class II TEs. The remaining 3.51% are unknown, unspecified, low complexity rRNA and simple repeats. This suggest that the composition of repeat elements within the genomes of the same AG are more likely to be similar compared to those from different AGs and other pathogens (Figure 1). However, out of the known repeat elements (excluding Unknown and Unspecified repeat elements for fair comparison), Long Terminal Retrotransposon (LTR) from Class 1 TEs make up the highest number of repeat elements in the Draft AG1-IA (1.02%). LTR is also prevalent in China AG1-IA (4.5%) and AG8 (4.13%). Despite the variability shown in the composition of TE superfamilies between the three genomes, a similar trend was observed in superfamily dominance of known transposons applied and the mechanism of genome assembly (Table 1).

The identification of transposable elements (TE) in the five genomes was conducted using the same tool namely TransposonPSI, which was used to eliminate any variation arising from the utilization of different tools and methods. This tool allows for the identification of abundance, class, and superfamily assignment (Supplementary Table S1). Figure 1A provides TE superfamilies in the *R. solani* genome of different AGs in terms of copy number while Figure 1B provides values in percentage. TE proportion out of the whole genome size for respective studied genome was determined. Here we report that the Draft AG1-IA has 2.22% TEs, which is lower than TE in China AG1-IA (3.67%), AG1-IB (3.18%), and AG8 (2.8%). The lowest TE proportion was seen in AG3 (1.02%). Our analysis shows that *R. solani* genomes have lower TE content compared to typical fungal pathogens, which generally contain an average of 10–15% of repeat elements [35]. However, as a Basidiomycete, *R. solani* TE values fall within the reported average TE content range of 0.1% to 45.2% [36]. The low TE abundance in *R. solani* could be one of the strategies used to ensure genome stability. This assumption is made based on a previous study, where it was reported that both retrotransposon and host are able to affect one another [37]. The genomes remains stable even with large number of active elements due to the protein-coding and regulatory sequences in the genomes [38]. Further, genomes have a way of suppressing modifications imposed by TEs, and usually contain mechanisms to eliminate these active elements, thus minimizing its mutagenic effects. These mechanisms include RNA interference (RNAi) or methylation of repeat sequences thus resulting in the inactivation of the retrotransposon [39]. Amyotte et al. (2012) also reported that filamentous fungi adopt repeat-induced point mutation (RIP), to deal with repetitive sequences such as TEs. This has been observed in *Neurospora crassa* and *Verticillium* spp. [40].

Class I TEs are found in a significantly higher copy number than Class II across AG3, China AG1-IA, and our Draft AG1-IA. Most fungal pathogens have a higher content of Class I TEs compared to Class II with a few exceptions such as in *Fusarium oxysporum*. The greater representation of Class I TE is a common observation among filamentous fungi [41]. ‘Copy and paste’ transposition mechanism adopted by Class I TE favors genome expansion [32]. However, based on our findings, the increase in number of Class I TE does not directly correlate to the genome size. For example, China AG1-IA has 4.90% Class I out of 5.27% total repeat elements with a genome size of 36.94Mbp compared to AG8 with 4.13% Class I repeat elements with the largest genome size of 48.83Mbp among the five genomes (Figure 1). This shows that having a large number of Class I TEs does not necessarily translate into larger genome size. Losada et al. (2014) in their study of mitochondrial and nuclear genomes of AG3 reported that mobile elements influenced the mitochondrial genome but there was no mention involving genome expansion of the nuclear genome. Further it is believed through various processes within the pathogen including epigenetics, TE content is suppressed and therefore results in genome stability and lack of genome expansion [42].

Following the identification of TE abundance and its classification, the dominant superfamilies were further scrutinized. Class I Long Retrotransposons (LTRs) such as *Gypsy* and *Copia*, are the most dominant superfamilies found in all the genomes with a slight difference in AG3, which has hATs as the second highest subfamily of total TEs (19.68%). hATs is also found abundantly in AG8, although *Gypsy* and *Copia* still remain as predominant superfamilies (Figure 1A,B). *Copia* and *Gypsy* resemble retroviruses in terms of structure due to the presence of LTRs and internal open reading frames (ORFs) [43]. Both these TE subfamilies encode four protein domains including protease, integrase, reverse transcriptase, and ribonuclease H. Based on the presence and absence of certain encoded protein domains, *Copia* and *Gypsy* can be further subdivided into several lineages. The lineages for *Copia* are Sireviruses, Oryco, Retroit, TORK, and Bianca, while *Gypsy* are Errantivirus, Chromoviruses, Ogre-elements, and Metavirus [44].

The next most dominant superfamily in the Draft AG1-IA is LINE, a Class 1 TE (7.46%), which is comparable to AG1-IA, AG1-IB, and AG8 with higher proportions observed in AG3 (Figure 1). Following LINE, ltr_Roo from Class 1 TEs was also identified in the Draft AG1-IA. However, ltr Roo were found substantively after *Gypsy* and *Copia* in AG1-IA, AG1-IB, and AG8. The Draft AG1-IA lacks ISC1316, which constitutes about 1.03% and 0.31% in AG1-IA and AG8, respectively. The remaining superfamilies such as CACTA, mariner_ant1, mariner, helitronORF, Crypton, and unknown (lsb) have similar proportions across all the genomes (Figure 1A,B). Amyotte et al. (2012) and da Silva et al. (2020) observed similar profiles with high Copia, Gypsy, LINE, hAT, and Tc1/mariner-like transposons in surveyed Verticillium spp. and Colletotrichum spp. [40,45]. While dominant TEs remained the same, the subfamilies distribution varied. In Verticillium spp., a detailed study of the TEs provided some proof of their involvement in genome evolution and inter and intra-specific species diversification [40]. So, whether the number of TEs were high or low, these insertions were sufficient to generate diversification and variation in species and this enabled quick adaptation to niche host.

However, the definite cause of genome distribution of certain subfamilies for these repeat elements have not been clearly determined as some of the variation is simply due to chance, illegitimate recombination and unequal homologous recombination, which usually causes the removal of TE [46]. Determination of the cause for this variation would give a significant insight into the evolution of fungal genomes [44].

### 2.3. Signal Peptides

**Figure 2:**
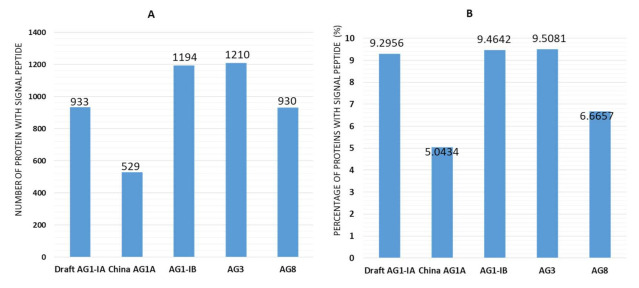
Secreted Proteins. (**A**) Number of proteins with signal peptides across five AGs of *R. solani*. (**B**) Percentage of proteins with signal peptide out of all predicted protein-coding genes across five AGs of *R. solani*.

Fungi produce secretomes that are actively transported out of the cell and play a vital role in fungal pathogenicity [47]. Therefore, all proteins were analyzed for the presence of signal peptides to identify potential proportion of secreted proteins in the Draft AG1-IA and the other four AGs classes. Figure 2 shows the number of potential secreted proteins as signified by the presence of signal peptides through SignalP 4.1 analyses. Essentially, these predicted secreted proteins can provide basis for further identification of effector candidates. These effectors are key players in the plant microbe interaction. Predicted secreted proteins in Draft AG1-IA genome constitutes among the highest (9.29%; 933 proteins) out of all the predicted protein, AG3 (9.51%; 1210 proteins), and AG1-IB (9.46%; 1194 proteins). Lower proportions of predicted secreted proteins were observed in AG8 (6.67%; 930 proteins) and China AG1-IA (5.04%; 529 proteins). A difference of 4.26% in secreted proteins was observed between the Draft AG1-IA and China AG1-IA. Various secretory proteins have been linked with pathogenic function including inducing cell death, necrosis, and plant immune system [48]. Different effectors that cause cell death and trigger immune systems of host plant have been identified in different AGs of *R. solani*. This includes AGLIP1 in AG1-IA, RsLysM in AG2-2IIIB, and RsIA_NP8 in AG8 [49,50,51]. AGLIP1 plays a significant role in the inhibition of basal defense and induces necrosis in hosts [50]. Similarly, RsIA_NP8 in AG8 induced cell death and triggered ROS burst in *N. benthamiana* [49]. On the other hand, AG2-2IIIB employs LysM effector to break down chitin-triggered immunity, which is mostly observed with hemibiotrophic pathogens [51,52]. This indicates that different effectors may be utilized by different AGs to perform similar or different functions in virulence.

Difference in predicted secreted protein profiles between Draft AG1-IA and China AG1-IA suggests that despite belonging in the same AG, strain variation may have contributed to the difference in predicted effector candidates observed as a result of local adaptation to host [53]. Draft AG1-IA and China AG1-IA may employ specific strategies and rely on key dedicated secreted proteins regardless of the amount to colonize the host plant [19].

### 2.4. CAZy Distribution Analysis

To penetrate into the plant cell wall that acts as the first line barrier for the plants, phytopathogens employs carbohydrate active enzymes (abbreviated as CAZy in this paper) for successful establishment of infection. Based on the CAZy.org database, the proportion of protein-coding genes that code for CAZy were assigned to their family and subfamily classes [19]. CAZy distribution in the five studied genomes were analyzed (percentage of the total number of CAZy to the total number of proteins in their respective genomes) to decipher the infection strategy adopted by *R. solani* through its enzymatic actions. Our analysis of CAZy showed a total of 315 CAZy identified in the Draft AG1-IA, which accounts for 3.14% from the total predicted protein coding genes (Table 3). This is close to the percentage of CAZy present in AG3 (3.21%), and AG1-IB (3.05%), while AG8 and AG1-IA recorded lower content of CAZy at 2.00% and 1.16%, respectively.

Overall, the predicted CAZy in *R. solani* are relatively low when compared to other pathogenic fungi despite having the ability to infect wide host range [1]. This implies that pathogenic fungi may have specific adaptations to their host type, complexity of host cell wall, in addition to specific infiltration and colonization strategies [54]. Secreted proteins, enzymes of primary and secondary metabolism, and transporter proteins show the varied strategy of *R. solani* AGs to maintain their necrotrophic lifestyle [19]. This variation in strategies to degrade plant cell wall is also observed by a previous study where comparisons were made between different strains of a basiodiomycete fungi, *Ustilago maydis* [55]. Therefore, it is possible that this is a trend with basidiomycete fungi.

**Figure 3:**
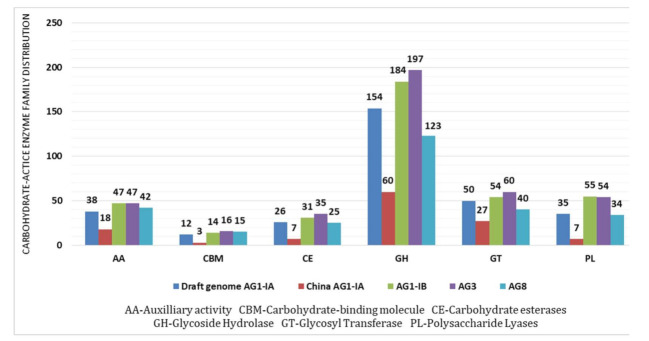
Secreted protein in the five AGs. The above figure provides the Carbohydrate-active Enzymes (CAZy) family distribution. AA—Auxiliary Activities, CBM—Carbohydrate-binding Molecule, CE—Carbohydrate Esterases, GH—Glycoside Hydrolase.

To provide a deeper understanding on how CAZy contributes to the pathogenicity of *R. solani*, a closer look into CAZy families was conducted. The distribution of CAZy families is divided into six families according to sequence-based classification in CAZy database [56]. The overall distribution of each CAZy family indicates Glycoside Hydrolase (GH) as the most abundant class of CAZy in Draft AG1-IA (1.53%) followed by Glycosyl Transferase (GT) (0.5%), Auxiliary Activities (AA) (0.38%), Polysaccharide Lyase (PL) (0.35%), Carbohydrate Esterase (CE) (0.26%), and Carbohydrate-binding modules (CBM) (0.12%) (Figure 3).

A similar trend was observed in China AG1-IA with slight variations in proportions of each CAZy family. GH dominance is also recorded in the other genomes. The dominance of GH is also a typical observation for diverse fungi along with CE [57]. In the Draft AG1-IA, GH is highly represented by GH16 (β-glucanases), GH5 (cellulases), GH13 (α-amylase), and GH28 (polygalacturonases) (Table 4). Majority of the GH classes in our Draft AG1-IA were from the GH28 family, which were mostly polygalacturonases (PG) associated with hydrolysis of galacturonic compound found in pectate [58]. This naturally results in efficient degradation of pectin backbones in plant cell walls assisted by PL families [59]. GH16 and GH5 were prevalent in all the other AGs. GH28 were also found in abundance in AG1-IB, AG3, and AG8, which is lacking in China AG1-IA that has copious amounts of GH13 similar to the Draft AG1-IA. The GH5, GH13, and GH16 (endoglucanase, exoglucanase, and β-glucosidase) families are involved in the degradation of cellulose, a component of plant cell wall [60]. These enzymes therefore aid in the initiation of the infection process in *R. solani* [61]. A transcript profiling study on AG1-IA revealed that, GH28, GH5, CE5, and PL4 was upregulated after 48 h of infection [62]. GH28 are pectinases utilized by pathogens to acquire host nutrients. CE5 are cutinases that were shown to aid in the direct penetration of *P. brassicae* into host surface through an enzymatic mechanism [63]. PL4 is a pectinolytic enzyme that is common in necrosis-causing pathogens [64]. Collectively, these components contribute towards plant cells necrotrophy.

Meanwhile, for Glycosyltransferases (GT), the most common families found in the Draft AG1-IA are GT2 (cellulose), GT1 (uridine diphosphate-dependent), and GT4 (hemicellulose). Likewise, GT2 and GT4 were abundant in the rest of AGs while GT1 was frequently found in all AGs except China AG1-IA. GT2, which is the most abundant, is a chitin synthase that is essential in chitin synthesis in fungal cell wall [65]. This results in the rigidity of the cell wall barrier, shape, biological metabolism, and interaction with host defense mechanisms, which results in the protection of the fungi from its environment (Table 4) [66].

Pectin lyases or pectate lyases are produced mainly by fungi to degrade the pectin component in plant cell walls [59]. Our analysis showed PL1 (pectate lyase, exo-pectate lyase, pectin lyases, pectin lyases), PL3 (pectate lyase), and PL4 (rhamnogalacturonan endolyase) are found in all the genomes except China AG1-IA, which has no PL3. The activity of these three families (pectate lyase, exo-pectate lyase, pectin lyase, and rhamnogalacturonan endolyase) provide the lytic enzymes that degrade the pectin component of the plant cell wall (Harholt, 2010). PL1 is also closely associated with GH28 to enhance pectin lysis. This naturally aids the infection process by pathogenic fungi in breaching the host physical layer (Table 4) [67].

On the other hand, for AA family, AA9 (lytic cellulose monooxygenase), AA5 (galactose oxidase, glyoxal oxidase, and alcohol oxidase), and AA1 (laccase) are found in the Draft AG1-IA, as observed in other AGs, with the exception of China AG1-IA that lacks AA9 (Table 4). AA1 is a multicopper oxidase with activities including laccase, ferroxidase, and laccase-like multicopper oxidase while AA5 is a copper radical oxidase [68]. Both these subfamilies are known to have the ability to oxidize a wide range of compounds including lignin [69]. AA9 (formerly GH61), a lytic polysaccharide monooxygenases (LPMOs), acts directly on cellulose and works synergistically with other cellulose enzymes to enhance the hydrolysis of cellulose producing substrates that are accessible to other CAZy [70] (Table 4). Transcriptomic profiling of *R. solani* in a recent study indicated that AA9 transcripts, which were present in high copy number, were found to be up regulated after inoculation. Particularly, the PMO1 (polysaccharide monooxygenases-1) group of AA9 transcripts showed the highest expression level as compared to other groups. This implied that AA9 plays an important role in the pathogenicity of AG1-IA [62].

CE8 (pectin methylesterase), CE5 (cutinase), and CE4 (chitin deacetylase) were dominantly found in Draft AG1-IA as well as other AGs except China AG1-IA where only CE4 and CE8 were highly represented. CE5 includes cutinases that catalyzes the hydrolysis of cutin, a hydroxy fatty acid that plays a role in plant protection layer. Thus, the breakdown of the host protection layer would facilitate the infection process by *R. solani* [71]. CE8 has pectin methylesterase, which catalyzes the hydrolysis of homogalacturan chains, one of the three pectin structural elements besides rhamnogalacturonan-I and galacturonans [38]. As pectin is one of the major complex components in plant cell wall, the high presence of CE8 family would enhance the infection efficiency by *R. solani* (Table 4) [57]. Collectively, these enzyme arsenals are there to protect the fungi and to assist with the infiltration of pathogen into host. Meanwhile, both CE and CBM were the least abundant CAZy classes in all five studied genomes. Based on our analysis, CBM13 (lectin-like proteins) and CBM1 (binds chitin and cellulose) are the two prevalent families in Draft AG1-IA and other AGs except for China AG1-IA, which only has CBM13. Transcriptomic analysis on AG1-IA revealed that the upregulated differentially expressed genes (DEGs) during infection in rice belongs to GH, CBM, and GT families and none from CE family [19]. The transcript analysis supports the findings observed by genome informatics.

The CAZy family variation showed highly similar representation for all studied genomes except slight variation recorded in GH and CBM and lack of these CAZy family representation in AG1-IA This shows that despite belonging to the same anastomosis group, the employment of CAZy to infect host plant may vary in the *R. solani* isolates with pathogenicity strategy that is possibly customized to different host and host resistance level [72]. Different AGs may employ different cohorts of carbohydrate-active enzymes or key effectors to initiate infection as their strategy as opposed to relying on large numbers of enzymes. For example, this is demonstrated by the underrepresentation of CAZy families and number of CAZy in China AG1-IA, which is supported by the original study of this AG. Zheng et al. (2013) deduced that China AG1-IA only needs key pathogenic GH and genes and does not rely on a large number of CAZymes and secondary metabolites during host infection. China AG1-IA genome encodes diverse sets of secreted proteins and enzymes with enriched sets of cell wall-degrading genes such as pectinase, xylanase, and laccase. The transcript profile showed that GH72, GH5, GH13, and PL4 marked a significant role in infection where these enzymes were recorded at high levels at different stages of infection in *R. solani* [19]. Meanwhile, Wibberg et al. (2016) reported that AG2-IIIB has specific pattern of abundance and distribution of CAZy, which includes diverse GH43 group (Glycoside Hydrolase), abundant PL1 (Polysachharide lyase), and enriched CE12 proteins (Carbohydrate esterases) compared to other AGs of *R. solani* and other fungal pathogens [26]. This further substantiates the conclusion that all AGs do not have the same composition of CAZy. It may differ according to the host range, strain, and complexity of the genome [26].

### 2.5. Pathogenicity Genes Distribution and the Impact of TEs on Pathogenicity

**Figure 4:**
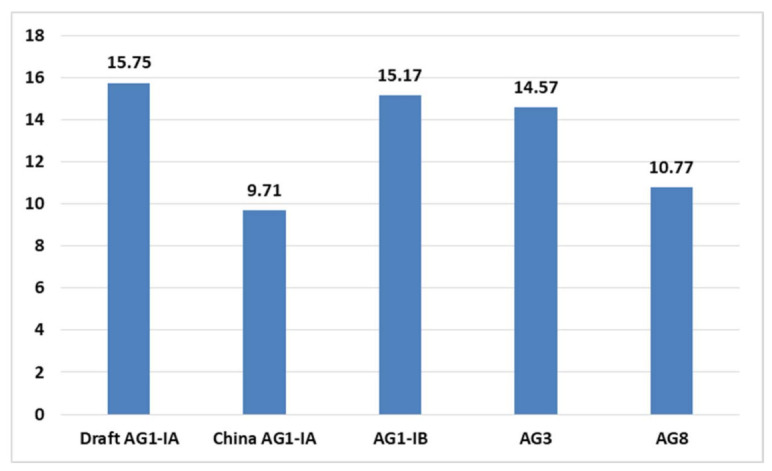
Percentage of pathogenicity genes out of total protein coding genes in the different AG genomes.

In order to mount a successful infection on host, phytopathogens rely on a group of genes crucial for disease development known as pathogenicity genes. Exploring different pathogenicity genes that exist within a phytopathogen can provide valuable insights on the host pathogen interaction during infection process and in the long run suggest target genes for disease control. Pathogenicity genes are often associated to processes such as appressorium formation, melanin biosynthesis, cell wall (host) degradation, production of fungal toxins, and signaling genes. Our analysis on pathogenicity genes distribution indicates that broad host range pathogens do not have high distribution of pathogenicity genes as demonstrated by AG8 (10.77%) (Refer Table 5). In contrast, the Draft AG1-IA, which can be categorized as a narrow host-range pathogen recorded the highest percentage of pathogenicity genes (15.75%). Interestingly, however, despite belonging in the same anastomosis group China AG1-IA, recorded the lowest percentage at 9.71% as shown in Figure 4.

**Figure 5:**
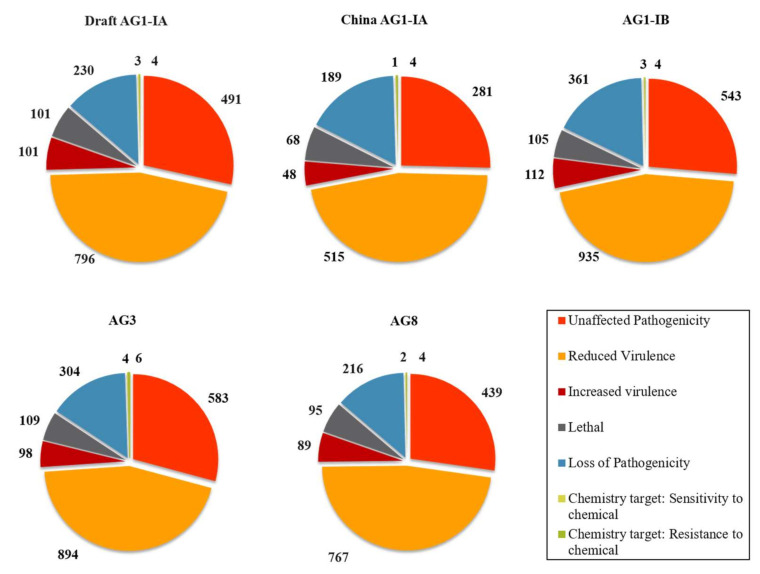
Distribution of pathogenicity genes assigned to characteristic of mutant phenotype across five different AGs of *R. solani*.

The pathogenicity genes are also assigned to its mutant phenotype characteristics. Our findings show that the most dominant assignment of mutant phenotypes belong to “reduced virulence” category (Figure 5). This implies that most predicted genes are associated with determining the severity spectrum of infection, defined by virulence rather than the cause of infection as implied by the term pathogenicity. As observed in AG2-IIIB, this category is also dominant and displays a range of virulence, which may also represent the saprophytic ability of this fungi [26].

Next, we explored the link between TEs and pathogenicity genes given previous observations where TEs were often found clustered near coding genes, influencing or altering their expression levels [36]. For instance in *Magnaporthe oryzae*, transposable elements were found to sit within 1 kb distance from genes encoding secreted proteins [73]. TEs were also observed to induce the expression of its neighboring genes. In addition, the insertion of a part of LTR retrotransposon into the promoter region of a gene coding for MFS1 transporter overexpressed this gene. This resulted in enhanced fungicide resistance [74]. Effector genes with actively transposing TEs, in lineage-specific (LS) rich regions, were overrepresented after infection in *Verticillium dahliae* transcriptome. It is likely that the chromosomal rearrangement of the TE drives the virulence of the pathogen [75]. Hence, observations were made on the physical distance between the pathogenicity genes and TEs to observe any distinctive patterns.

**Figure 6:**
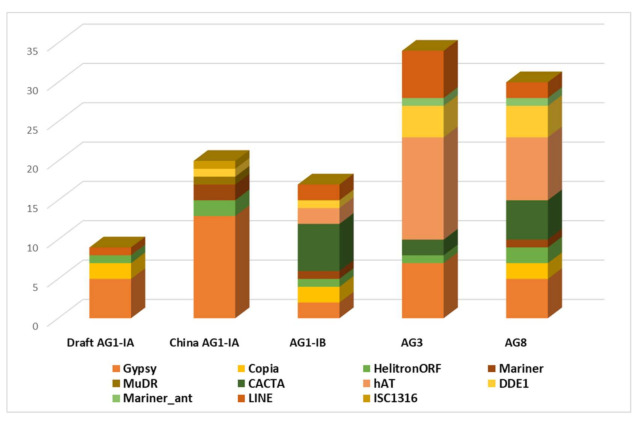
Proximity analysis. Distribution of TEs in vicinity with pathogenicity genes.

Figure 6 shows the distribution of TEs in the vicinity of pathogenicity genes. From the results, there are substantial occurrences where TE is inserted in proximity with the pathogenicity genes (within 5000 bp). The most frequent insertions were found in AG3 (109), AG8 (91), followed by China AG1-IA (67), AG1-IB (46), and Draft AG1-IA (31) (Supplementary Table S2). The closest insertion of TE was found 22 bp from pathogenicity gene in AG3 and 180 bp from a gene in the Draft AG1-IA.

Due to its abundance in the genomes, *Gypsy* is overrepresented as the TE superfamily that is found in the vicinity of pathogenicity genes. In AG3, however, this is replaced by haT. In *Coccidioides immitis*, genes that reside within 1 kb to a *Gypsy* or hAT transposon had lower expression [76]. This shows that the proximity of TE to the genes affects the expression of the said gene. MAGnaporthe GYpsy-like element (MAGGY) contributed to genome instability in *Magnaporthe oryzae* upon imposition of stress [77]. Other TE superfamilies were not overrepresented in proximity cases. Some superfamilies are more “successful” than others in inserting themselves near the pathogenicity genes, though factors driving the insertion and selection remains unknown. For example, ltr-roos are found abundantly in AG1-IA, AG3, and AG8 but they are not found to be in vicinity to pathogenicity genes annotated within these genomes. Meanwhile, CACTA is found more in AG1-IB and AG8 despite its relative low abundance in comparison to other superfamilies.

However, in this study, the number of TEs in vicinity of pathogenicity genes is low. In other phytopathogens like *Magnaporthe* spp and *Verticillium* spp., it has been reported that TE distribution can either be patchy or as hotspots. These distribution profiles may result in the low incidence of TE being inserted next to a pathogenicity gene. While the TE may not be seated next to a pathogenicity gene, they may play a role in modulating genome architecture allowing for quick adaptation to niche host [28,40]. Further it is more than likely that *R. solani* may depend on other compounds and enzyme such as secondary metabolites including oxidoreductase [78], O-methyltransferase [79], and virulence and effector proteins such as glucanases, cutinase, and pectin lyase [80] to maintain a pathogenic lifestyle, as opposed to the influence of TE insertion near pathogenicity genes.

### 2.6. Single Copy Orthologs

Comparative analysis done on the five *R. solani* genomes provided a platform to understand variation in genomic content and its connection to host infection strategies. Nonetheless, it is also vital to identify core genes within the *R. solani* genome necessary for survival as fungal pathogens, through orthologous gene analyses in the five genomes. The general statistics of the orthologous groups identified are presented in Table 6 and detailed statistics attached as Supplementary Table S3.

From our findings, a total of 3936 core orthologs (genes present in all five studied genomes) were identified and out of these, 2758 are classified as single copy orthologs (Table 6). This number represents 27.5% of the total protein coding genes identified in Draft AG1-IA. These core orthologs were then characterized by their gene ontologies to gain insight on the most vital biological processes, molecular, and cellular components in *R. solani*, regardless of their membership in different anastomosis groups.

**Figure 7:**
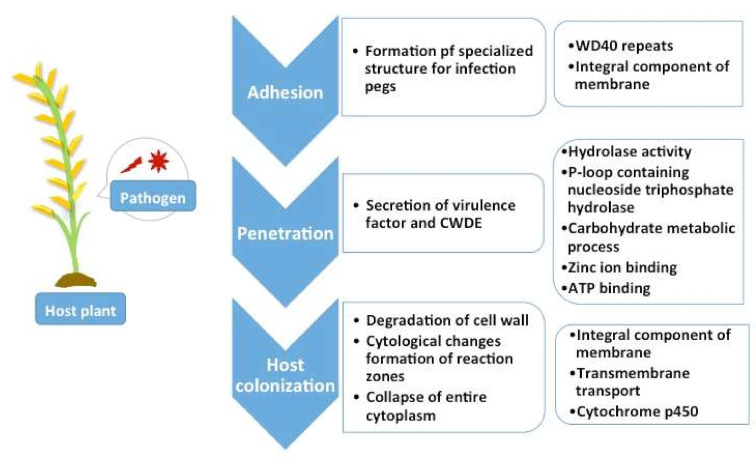
Phases of infection and associated dominant ontologies identified in *R. solani*.

The most dominant gene ontologies for biological processes, molecular functions, and cellular components categories for studied *R. solani* genomes are presented in Table 7. These most dominant ontologies are believed to be necessary for survival of the *R. solani* as a pathogen. Figure 7 shows how previously identified dominant ontologies (Refer Result Section—Genome Annotation of *R. solani* Draft AG1-IA & Comparative Genome Assembly Statistics) and those characterized as core orthologs infer possible roles and connection to the predicted necrotrophic lifestyle of *R. solani* AG1-IA. This is divided into three processes, mainly adhesion, penetration, and host colonization.

For phase 1, infection is initiated through the adhesion of spores and germ tube production on host surface. Proteins containing WD40 repeat promoted the stability of the adhesion protein complexes. Upon contact with host structure, Rhizoctonia spores adhere to the surface and initiate specialized hyphal structure for penetration into plant tissue. These structures are dependent on host specificity and will form infection cushions on host. Furthermore, WD40 has been directly implicated in pathogenesis and sexual reproduction in homothallic fungi [81]. AG1 to AG4 of *R. solani* are homothallic organisms.

In phase 2, the infection pegs promote penetration of host cell walls. The host–fungal interaction results in the secretion of extracellular enzymes (pectinase, pectin lyase, cellulose, and phosphatase) from the fungus and host exudates from the plant. This initiates degradation of plant cell wall and initiates penetration. *R. solani* through the deployment of a wide range of plant cell wall degrading enzymes (PCWDEs) such as glycoside hydrolases cleave the glycosidic bond in plant cell walls. Pathogenic fungi such as *M. grisea* secretes glycosides hydrolase, polysaccharides, and esterase to degrade plant cell walls, form necrotic lesions and for conidiogenesis [82,83]. Both families involve the common P-loop nucleotide triphosphate hydrolase family that provides energy for reactions or motion. In yeast, P-loop nucleotide triphosphate hydrolase is associated with a role in signal transduction, kinases, transferases, and as an energy provider [84].

Next, in the colonization phase, degradation of cell wall, cytological changes, formation of reaction zones, plasmolysis, and the collapse of entire cytoplasm results in breached barrier by the pathogen. Cytochrome P450 is a monooxygenase that catalyzes the transfer of oxygen to cellular substrates in fungi [85]. The enzyme produced by cytochrome P450 genes break down various molecules and are involved in the biosynthesis of primary, secondary, and toxic metabolites (detoxification) [86,87,88]. Cytochrome P450 is involved in the biosynthesis of ergosterol, an essential integral component of fungal membrane for structural integrity and permeability [89]. Cytochrome P450 also detoxifies xenobiotic including environment pollutant and antifungal compounds from the host defense system thus enabling pytopathogenic fungi to not just survive harsh conditions but to also aid in the infection process [66].

**Figure 8:**
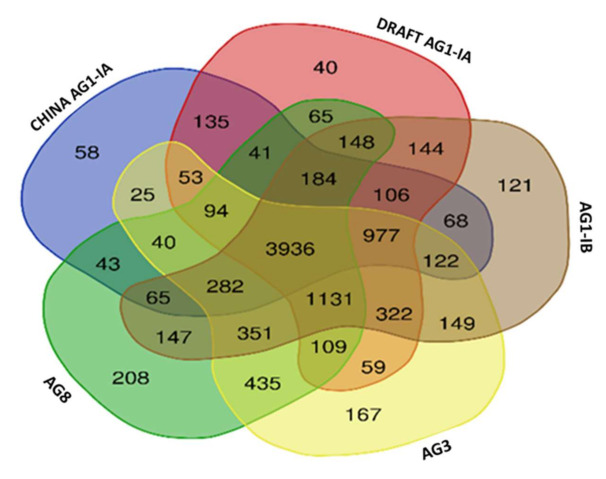
Venn diagram of the shared orthologous groups present in the five studied genomes of *R. solani*.

Next, the study also scrutinized the representation of the shared orthologs among the genomes. As presented in Figure 8, AG1-IB shares 144 most unique orthologs with the Draft AG1-IA. However, between China AG1-IA and the Draft AG1-IA there are 135 uniquely shared orthologs, followed by AG8 (65 unique shared orthologs) and AG3 (59). Based on an overall representation of the shared orthologs (including all types of shared orthologs i.e., shared between two genomes, three genomes, and so forth), we were able to observe that the Draft AG1-IA has the highest number of shared orthologs with AG1-IA (3017 shared orthologs) compared to AG1-IB (3004 shared orthologs) followed by AG-3 (2766 shared orthologs) and AG8 (2620). We also observed some shared unique orthologs of AG1-IA, present in both Draft AG1-IA and China AG1-IA. These orthologs can be classified as host-specific genes that make up the core genes for host infiltration in *R. solani*.

### 2.7. Synteny Analysis

Syntenic blocks represents genomic regions covering a number of genes that are orthologous and co-arranged with other mapped genome [90]. This estimated synteny between the genomes at scaffold level may be utilized for complete genome assembly in future.

Our synteny analysis revealed that the conserved blocks are considerably fragmented. Syntenic blocks also called locally collinear blocks (LCBs) are identified conserved regions among the studied genomes. Cumulatively, 2272 core LCBs are conserved in all five genomes studied and represents 12.9% out of a total 17,610 blocks identified. This spans almost 24.2Mbp in length. Most of these blocks are not considerably long in length, where even the largest LCB, LCB_1448 only represents ~1% in length of the entire reference genome of AG1-IA and most of these blocks are <100 kbp in size. There are 151 blocks that are small with mean length of less than 1 kbp. The overall number and characteristics of LCBs identified are presented in Table 8 below and detailed statistics attached as Supplementary Table S4.

**Figure 9:**
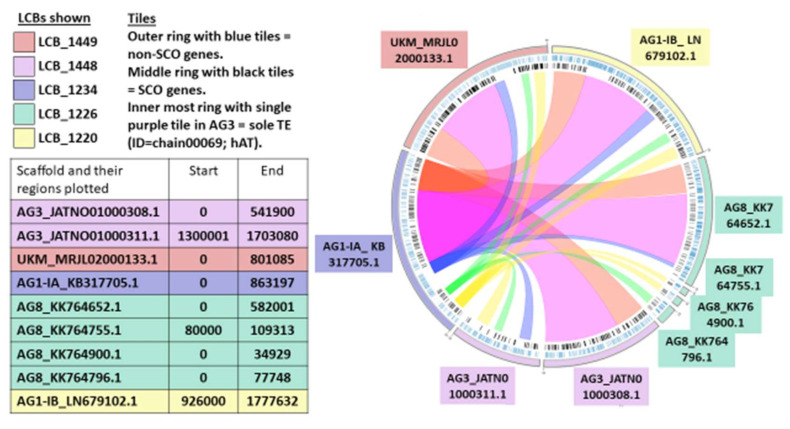
Circular Plot of five largest LCBs harbored in reference scaffold mapped to the regions in the other studied genomes of *R. solani*.

For better visualization of the LCBs, a reference scaffold, AG1-IA_KB317705.1 containing the five largest core blocks (LCB_1449, LCB_1448, LCB_1234, LCB_1226, LCB_1220) was mapped to other regions in the remaining four studied genomes as presented in the circular plot in Figure 9.

**Figure 10:**
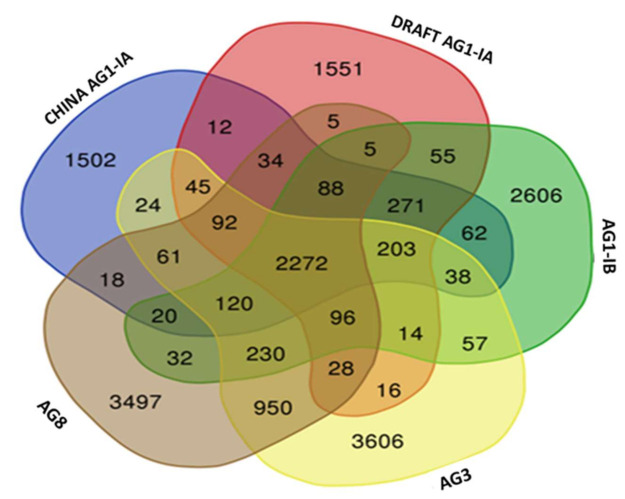
Venn diagram of the shared LCBs present in the five studied genomes of *R. solani*.

**Figure 11:**
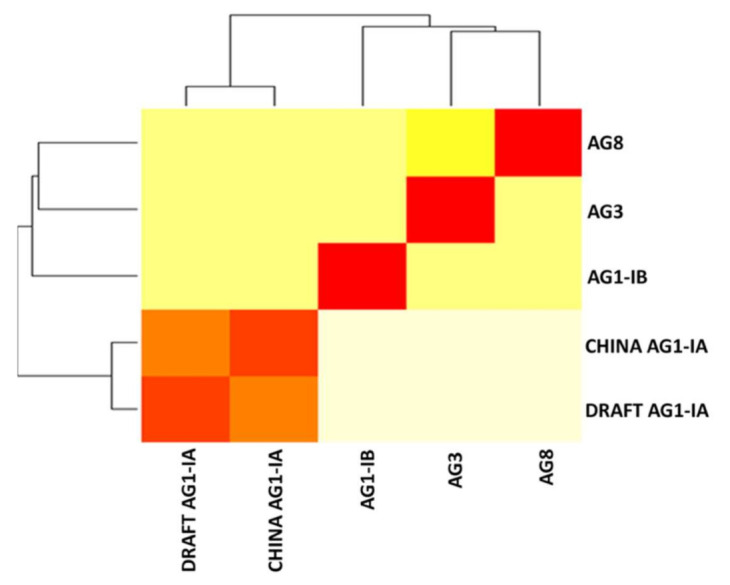
Genomic distance of five *R. solani* genomes in heatmap visualization.

The same trend is seen for shared locally collinear blocks (LCB) as observed in the Orthologous Groups, where more unique LCBs were shared between AG1-IB (55 blocks), of cumulative size of 49,479 bp in comparison with Draft AG1-IA and China AG1-IA, with 12 blocks only at 18,935 bp as shown in the Figure 10 below. Figure 11 shows the heatmap connectivity between the genomes. Similarly, this supports the relationship between Draft AG1-IA to China AG1-IA followed by AG1-IB, AG3, and lastly AG8.

Previous studies have highlighted that there is widespread co-linearity or macro-synteny between AG8, AG1-IA, and AG3 based on sequence comparison [22]. A comparative genome analyses of four rice-infecting isolates of *R. solani* belonging to anastomosis group 1 showed high levels of synteny within the group (66.4–70.9%). This explains the high correlation in synteny between the Draft AG1-IA and China AG1-IA [91]. Losada et al. (2014) observed that the mitochondrial genome of AG3 were highly syntenic with AG1-IA and AG1-IB (>95%) where the sequence and order of the core mitochondrial genes were generally conserved with exception to accessory and non-coding regions [92]. It is believed that the regions of synteny will predominantly be shared regions of importance carrying core functions of the fungi, including reproduction and pathogenecity. Evolutionary relationship is bound to factor into the level of synteny observed. The more closely related species are expected to share more syntenic blocks. As AG1-IB derives from AG1, this is perhaps why this AG shares the most blocks with AG1-IA. As presented in Table 8, most syntenic blocks represented by the LCBs are short. This may be attributed to the fragmentation at the genome assembly level of all five studied genomes, as seen in the N50 values of their assembly.

## 3. Materials and Methods

### 3.1. Gene prediction & Genome Annotation

Gene prediction for our genome assembly *R. solani* Draft AG1-IA was conducted de novo via Gene-Mark-ES fungal version and referenced against the *R. solani* AG1-IA from China. Gene content characterization was determined through blastp function by subjecting the protein-coding genes to non-redundant database analysis in the fungal division. The results were then filtered to 60% similarity and 80% query coverage. Each of these genes was subjected to Gene Ontology annotation to identify the molecular functions, biological processes, and cell components related to these genes. Further, functional analysis via InterPro was conducted to retrieve the domains and families assigned for these genes. Characterization through Gene Ontology and InterPro was filtered at 0.005 e-value cutoff.

### 3.2. Repeat Elements Characterization

Following assembly, repeat elements identification was achieved through a combinatorial approach of de novo and homology-model based analysis. Draft AG1-IA assembly of *R. solani* 1802 K/B was analyzed for repeat characterization through RepeatScout, RepeatModeller, and Repbase to generate consensus repeat distribution. For the model-based, repeats were characterized in Draft AG1-IA based on fungal repeat library available in RepBase. For de novo identification of repeats, no reference library was used, and our Draft AG1-IA was used for self-training. The de novo repeat library database was generated using input Draft AG1-IA. The tools used were RepeatScout and RepeatModeller and an output file was provided as input to RepeatMasker to mask repeats. Consensus repeats were reported by combining repeats identified in all the above three steps using buildSummary in RepeatMasker.

### 3.3. TE Identification

The tool used to identify TE is TransposonPSI, which adopts PSI-Blast in finding similarity search against transposon ORF profiles. The settings were at default where nucleotide sequence of the assembly file was used as input. The results were divided into outputs, which are All Hits Chain and Best Per Locus. These results were gathered based on collection of high-scoring pairs with homology to collection of TE ORFs, which was then chained together based on collinear position. The distribution of TE will be analyzed by looking at All Hits found in a chain, meanwhile Best Per Locus results were manipulated for use in the TE position analysis in the later section.

### 3.4. Pathogenicity Gene Distribution

Protein-coding genes of all studied genomes was used as query in pathogenicity gene identification by mapping them against curated PHI-base database, which compiles effector, pathogenicity, and virulence genes [93]. The resulting homologs will then be further filtered to e-values of 1 × 10^−3^, 80% query coverage, and 35% similarity, which is deemed to be appropriate cutoff parameters for reliable homology and stringency.

### 3.5. Proximity Analysis: Link to TE Influence in Pathogenicity

The proximity analysis was set within a defined distance window where pathogenicity genes (PG) were found upstream or downstream of transposable elements (TE). The computed distance between TE-PG and the proximity scale was set within 1 kb (possible distance to give impact in altering gene expression) and 5 kb (to explore more possibilities, lower stringency), and analyzed via Bedtools Version 2.28.0 (Swiss-knife). The query data used here were all the TEs retrieved from TransposonPSI (at Best Per Locus) and filtered pathogenicity genes obtained for all the five (5) genomes studied.

### 3.6. Signal Peptides

Secreted protein distribution analysis was conducted using protein-coding genes as input queries to SignalP 4.1. This tool allows the identification of the proteins with signal peptides hence signifying secreted protein out of membrane. Proteins found containing signal peptides can be regarded as secreted proteins. All predicted protein containing signal peptide were crosschecked with proteins containing transmembrane helices (identified by TMHMM2.0 algorithm) before classifying them as secreted proteins.

### 3.7. Carbohydrate Active Enzymes (CAZy)

To understand the specific functions of secreted proteins in relation to the infection process, protein functions were predicted against CAZy database. This was achieved by mapping the predicted genes to the retrieved CAZy database and the results were further filtered to 60% query coverage and 50% similarity. dbCan database was retrieved which compiles carbohydrate active enzymes sequences and allows full-length annotation of CAZy family classification as featured in CAZy database with extra features of including sub family annotation [94]. Protein-coding genes of all the 5 genomes were used as input queries. Following files were retrieved from the web server for use as mapped databases and annotation purposes.

- CAZyDB.07202017.fa.
- CAZyDB-ec-info.txt.
- CAZyDB-ec-info.txt.07-20-2017.

### 3.8. Single Copy Ortholog Analysis

Single Copy Ortholog analysis was achieved via OrthoMcl. Protein sequences from different AGs were first compiled, followed by clustering by orthologous groups identified amongst the genome by all-to-all NCBI blastp. All protein sequences were first filtered to obtain good quality proteins (>33 aa and <30% stop codon). The results were then filtered to obtain single copy orthologous groups.

### 3.9. Synteny Analysis

Syntenic blocks were located by aligning the five retrieved genome assembly files. This is achieved via an assistive alignment tool, ProgressiveMauve, which enables identification of locally collinear blocks (LCB) using default parameters.

## 4. Conclusions

This work provides a comprehensive comparative analysis of *R. solani* from different anastomosis groups through the lens of our own sequenced and assembled genome, Draft AG1-IA, the second AG1-IA genome to be sequenced and assembled after the Chinese isolate. Our exploration of the TE landscape between the AGs showed no direct correlation to host range between the AGs. The percentage of TE in *R. solani* were low and this is possibly a mechanism by which it remains stable while utilizing specific arsenals targeted to their specific host for optimum colonization and minimal utilization of energy. *Gypsy*, *Copia*, and hAT were the main TEs observed and have been implicated in genome evolution and inter and intra-specific species diversification. While TE was observed next to pathogenicity genes, the distribution was low. While patchy and hotspot genome distribution of TE is common in phytopathogens, these elements may still be involved in adaptation to niche host.

Our genomic profiling shows that each *R. solani* genome irrespective of its AGs adopts unique specific strategies for infection, as observed in the variation seen at genomic level despite similarities in dominance of Glycoside Hydrolases. In addition, as part of its necrotrophic lifestyle, this fungal pathogen relies on the secretion of molecules especially cell-wall degrading enzymes as part of its virulence and pathogenicity strategy. Furthermore, based on the comparative profiling done on pathogenicity gene distribution, it is suggested that the number of pathogenicity genes may play little or no role on host range in *R. solani*. Therefore, we may conclude that each isolate of *R. solani* may use its specific arsenal to infiltrate and colonize the host i.e., the core arsenal. It is likely that the necrotrophic lifestyle of *R. solani* AG is controlled by the plethora of enzymes such as GH compared to the distribution and density of TEs. However, this will require further study.

## Figures and Tables

**Table 1:** Comparative genome informatics of different AGs of *R. solani*. The assembly size of each AGs shows that our Draft AG1-IA has the smallest size compared to other AGs while AG3 (51.71 Mbp) is the largest.

Genome	Draft AG1-IAT	China AG1-IA	AG1-IB	AG3	AG8
**Reference**	Nadarajah et al. 2017	Zheng et al. 2013	Wibberg et al. 2012	Cubeta et al. 2014	Hane et al. 2014
**Host range**	Isolated from rice	Rice, maize, soybean	Lettuce, bean, cabbage and carrot	Solanacae family	Cereals, brassicas, legumes
**Sequencing Method**	Illumina Mi Seq	Illumina Genome Analyser II	Illumina Mi-Seq	Sanger (4-, 10-, and 40-kb insert sizes), GS-FLX 454 (fragment and 20-kb mate pair)	Illumina
**Assembly method**	Velvet Medusa	SOAP denovo 1.05	Newbler Assembly version 2.8	Celera Assembler 5.1, CLC	SOAP denovo 1.05, SSPACE 2.1, GapCloser 1.2
**Assembly size (Mbp)**	28.93	36.94	42.80	51.71	48.83
**Accession number**	GCA_001899475.2	GCA_000334115.1	LN679100:LN679996	GCA_000524645.1	GCA_000695385.1
**N50**	420,418	474,500		25,869	
**GC content (%)**	46.99	47.61	48.04	48.41	48.83
**Number of scaffold**	1517	2648	897	326	861
**Protein coding genes**	10,037	10,489	12,616	12,726	13,964
**Repeat Elements (%)**	4.86%	5.27%	-	-	10.03

**Table 2:** Percentage of repeat elements in Draft AG1-IA, China AG1-IA, and AG8 and the different tools used for repeat identification.

Genome	Tools	% of Repeat Elements	Dominant Repeat
Draft AG1-IA	RepeatScout, RepeatModeller, RepeatMasker and RepBase	4.86	Long Terminal Retrotransposon (Gypsy)
China AG1-IA	PILER, RepeatScout, RepeatMasker and RepBase	5.27	Long Terminal Retrotransposon (Gypsy)
AG8	RepeatScout, CAP3, RepeatMasker and RepBase	10.03	Long Terminal Retrotransposon (Gypsy)

**Table 3:** The composition of carbohydrate active enzymes (CAZy) and the percentage of CAZy from total number of protein coding genes in the respective genomes.

Anastomosis Group (AG)	Total CAZy	Total no. of Protein-Coding Genes	Percentage of CAZy Out of Protein Coding Genes (%)
**Draft AG1-IA**	315	10,037	3.138388
**China AG1-IA**	122	10,489	1.163123
**AG1-IB**	385	12,616	3.05168
**AG3**	409	12,726	3.213893
**AG8**	279	13,952	1.999713

**Table 4:** The copy number of the members in each CAZy sub-family of *R. solani*.

AGs	Draft AG1-IA	China AG1-IA	AG1-IB	AG3	AG8
**Glycoside** **Hydrolase** **(GH)**	GH16 (15)	GH13 (6)	GH28 (25)	GH28 (21)	GH5 (17)
	GH5 (14)	GH16 (5)	GH16 (20)	GH 16 (16)	GH16 (13)
	GH13 (11)		GH5 (15)	GH5 (16)	GH28 (9)
	GH28 (10)				
**Glycosyl** **Transferases** **(GT)**	GT2 (12)	GT2 (8)	GT2 (12)	GT2 (11)	GT2 (9)
	GT4 (6)	GT4 (4)	GT4 (7)	GT4 (7)	GT4 (8)
	GT1 (6)	GT39 (3)	GT1 (6)	GT1 (5)	GT1 (4)
**Polysaccharide Lyases** **(PL)**	PL1 (15)	PL1 (5)	PL1 (23)	PL1 (25)	PL1 (14)
	PL3 (7)	PL4 (2)	PL3 (19)	PL3 (15)	PL3 (13)
	PL4 (7)		PL4 (9)	PL4 (10)	PL4 (3)
**Auxilliary** **Activities** **(AA)**	AA9 (11)	AA1 (5)	AA9 (11)	AA9 (13)	AA9 (11)
	AA1 (8)	AA5 (4)	AA1 (10)	AA5 (11)	AA5 (10)
	AA5 (6)	AA3 (3)	AA5 (9)	AA1 (11)	AA1 (8)
**Carbohydrate Esterases** **(CE)**	CE8 (8)	CE8 (5)	CE5 (9)	CE8 (11)	CE4 (7)
	CE5 (6)	CE12 (1)	CE8 (8)	CE4 (9)	CE5 (6)
	CE4 (5)	CE4 (1)	CE4 (7)	CE5 (6)	CE8 (6)
**Carbohdrate-Binding-** **Molecules (CBM)**	CBM13 (6)	CBM13 (3)	CBM1 (8)	CBM1 (9)	CBM1 (10)
	CBM1 (5)		CBM13 (5)	CBM13 (6)	CBM13 (4)
	CBM48 (1)		CBM50 (1)	CBM5 (1)	CBM50 (1)

**Table 5:** Host range classification of studied *R. solani* genomes.

Anastomosis Group (AG)	Host Range	Category	TE Abundance (%)	Assembly Size (Mbp)
**Draft AG1-IA**	Isolated from rice	Monocot	2.22	28.93
**China AG1-IA**	Rice, corn, sorghum, bean, soybean, turf grass, camphor, seedlings	Monocot-specific (Narrow)	3.67	36.94
**AG1-IB**	Rice, bean, soybean, leguminous woody plants, lettuce, hortensia, cabbage	Both monocot & dicot (Intermediate/Broad)	3.18	42.80
**AG3**	Solanaceae: Potatoes, tobacco, tomato, egg plant	Dicot-specific (Narrow/Intermediate)	1.02	51.71
**AG8**	Poaceae: Cereals, brassicas, legumes	Both monocot & dicot (Broad)	2.80	48.83

**Table 6:** Identification of orthologous groups.

Total Number of Proteins	59,588
**Number of Core Othologs**	3936
**Number of Single Copy Orthologs**	2758

**Table 7:** Characterization of core orthologs by dominant ontologies.

Cellular Component	Biological Processes	Molecular Function
Integral component of membrane	Translation	ATP binding
Nucleus	Transcription, DNA-templated	Metal ion binding
Cytoplasm	Transmembrane transport	DNA binding
Intracellular	Metabolic process	Zinc ion binding
Ribosome	Carbohydrate metabolic process	Hydrolase

**Table 8:** General locally collinear blocks (LCBs) statistics.

**Total Number of LCBs**	**17,610**
**Number of LCBs ≥ 1 kbp (mean)**	9697
**Number of LCBs ≥ 100 kbp (mean)**	46
**Number of Core LCBs**	2272
**Sum of mean length of Core LCBs (bp)**	27, 246, 585.6
**Number of Core LCBs ≥ 1 kbp (mean)**	2121
**Number of Core LCBs ≥ 10 kbp (mean)**	698
**Number of Core LCBs ≥ 100 kbp (mean)**	20

## Data Availability

The whole of genome of all studied *Rhizoctonia solani* can be downloaded from NCBI. A list of the accession numbers, related genome informatics and references can be found in Table 1. The authors confirm all supplementary data, code and protocols provided within the article or through supplementary data files. Four (4) supplementary tables are available with the online version of this article.

## Supplementary Materials

The following are available online at https://www.mdpi.com/1422-0067/22/4/2183/s1, Table S1: Transposable elements (TE) landscape of AG1-IA (UKM) which includes abundance, class and superfamilies assignment, Table S2: Distribution of TE and superfamilies inserted in proximity with the pathogenicity-associated genes within 5000 bp, Table S3: Single Copy Orthologs, Table S4: Synthenic Block.

## References

[1] Nagaraj B., Sunkad G., Pramesh D., Naik M., Patil M. (2017). Host range studies of rice sheath blight fungus *Rhizoctonia solani* (Kuhn). Int. J. Curr. Microbiol. App. Sci.

[2] García V.G., Onco M.P., Susan V.R. (2006). Biology and systematics of the form genus Rhizoctonia. Span. J. Agric. Res..

[3] Wibberg D., Jelonek L., Rupp O., Hennig M., Eikmeyer F., Goesmann A., Hartmann A., Borriss R., Grosch R., Pühler A. (2013). Establishment and interpretation of the genome sequence of the phytopathogenic fungus *Rhizoctonia solani* AG1-IB isolate 7/3/14. J. Biotechnol..

[4] Loan L., Du P., Li Z. (2004). Molecular dissection of quantitative resistance of sheath blight in rice (*Oryza sativa* L.). Omonrice.

[5] Pinson S.R., Capdevielle F.M., Oard J.H. (2005). Confirming QTLs and finding additional loci conditioning sheath blight resistance in rice using recombinant inbred lines. Crop Sci..

[6] Liu S., Hall M.D., Griffey C.A., McKendry A.L. (2009). Meta-analysis of QTL associated with Fusarium head blight resistance in wheat. Crop Sci..

[7] Jia L., Yan W., Zhu C., Agrama H.A., Jackson A., Yeater K., Li X., Huang B., Hu B., McClung A. (2012). Allelic analysis of sheath blight resistance with association mapping in rice. PLoS ONE.

[8] Liu W., Maurer H., Reif J., Melchinger A., Utz H., Tucker M., Ranc N., Della Porta G., Würschum T. (2013). Optimum design of family structure and allocation of resources in association mapping with lines from multiple crosses. Heredity.

[9] Kumar I.S., Cheah B., Nadarajah K. (2017). In silico identification and classification of disease resistance genes and defense-related genes against sheath blight from QTL qSBR11-1 in rice (*Oryza sativa* L.). Undergrad. Res. J. Integ. Biol..

[10] Kumar I.S., Zaharin N., Nadarajah K. (2018). In silico Identification of Resistance and Defense Related Genes for Bacterial Leaf Blight (BLB) in Rice. J. Pure Appl. Microbiol..

[11] Kumar I.S., Amran N.A., Nadarajah K. (2018). In silico Analysis of qBFR4 and qLBL5 in Conferring Quantitative Resistance Against Rice Blast. J. Pure Appl. Microbiol..

[12] Chanthran S.S.D., Cheah B.H., Nadarajah K.K. (2018). In silico analysis of disease resistance and defence-related genes for a major sheath blight qShb 9-2 QTL in rice. Malays. J. Microbiol..

[13] Walton J.D. (1996). Host-selective toxins: Agents of compatibility. Plant Cell.

[14] Friesen T.L., Zhang Z., Solomon P.S., Oliver R.P., Faris J.D. (2008). Characterization of the interaction of a novel Stagonospora nodorum host-selective toxin with a wheat susceptibility gene. Plant Physiol..

[15] Laluk K., Mengiste T. (2010). Necrotroph attacks on plants: Wanton destruction or covert extortion?. Arab. Book/Am. Soc. Plant Biol..

[16] Verwaaijen B., Wibberg D., Kröber M., Winkler A., Zrenner R., Bednarz H., Niehaus K., Grosch R., Pühler A., Schlüter A. (2017). The *Rhizoctonia solani* AG1-IB (isolate 7/3/14) transcriptome during interaction with the host plant lettuce (*Lactuca sativa* L.). PLoS ONE.

[17] Ghosh S., Kanwar P., Jha G. (2018). Identification of candidate pathogenicity determinants of *Rhizoctonia solani* AG1-IA, which causes sheath blight disease in rice. Curr. Genet..

[18] Cubeta M.A., Thomas E., Dean R.A., Jabaji S., Neate S.M., Tavantzis S., Toda T., Vilgalys R., Bharathan N., Fedorova-Abrams N. (2014). Draft genome sequence of the plant-pathogenic soil fungus *Rhizoctonia solani* anastomosis group 3 strain Rhs1AP. Genome Announc..

[19] Zheng A., Lin R., Zhang D., Qin P., Xu L., Ai P., Ding L., Wang Y., Chen Y., Liu Y. (2013). The evolution and pathogenic mechanisms of the rice sheath blight pathogen. Nat. Commun..

[20] Taheri P., Gnanamanickam S., Höfte M. (2007). Characterization, genetic structure, and pathogenicity of *Rhizoctonia* spp. associated with rice sheath diseases in India. Phytopathology.

[21] Ferrucho R.L., Cifuentes J.M., Ceresini P., García-Domínguez C. (2012). *Rhizoctonia solani* AG-3PT is the major pathogen associated with potato stem canker and black scurf in Colombia. Agron. Colomb..

[22] Hane J.K., Anderson J.P., Williams A.H., Sperschneider J., Singh K.B. (2014). Genome sequencing and comparative genomics of the broad host-range pathogen *Rhizoctonia solani* AG8. PLoS Genet..

[23] Anderson J.P., Sperschneider J., Win J., Kidd B., Yoshida K., Hane J., Saunders D.G., Singh K.B. (2017). Comparative secretome analysis of *Rhizoctonia solani* isolates with different host ranges reveals unique secretomes and cell death inducing effectors. Sci. Rep..

[24] Adhipathi P., Singh V., Meena S.C. (2013). Virulence diversity of *Rhizoctonia solani* causing sheath blight disease in rice and its host pathogen interaction. Bioscan.

[25] Sandoval R.F.C., Cumagun C.J.R. (2019). Phenotypic and molecular analyses of *Rhizoctonia* spp. associated with rice and other hosts. Microorganisms.

[26] Wibberg D., Andersson L., Tzelepis G., Rupp O., Blom J., Jelonek L., Pühler A., Fogelqvist J., Varrelmann M., Schlüter A. (2016). Genome analysis of the sugar beet pathogen *Rhizoctonia solani* AG2-2IIIB revealed high numbers in secreted proteins and cell wall degrading enzymes. BMC Genom..

[27] Thon M.R., Pan H., Diener S., Papalas J., Taro A., Mitchell T.K., Dean R.A. (2006). The role of transposable element clusters in genome evolution and loss of synteny in the rice blast fungus *Magnaporthe oryzae*. Genome Biol..

[28] Dean R.A., Talbot N.J., Ebbole D.J., Farman M.L., Mitchell T.K., Orbach M.J., Thon M., Kulkarni R., Xu J.-R., Pan H. (2005). The genome sequence of the rice blast fungus *Magnaporthe grisea*. Nature.

[29] Dong S., Raffaele S., Kamoun S. (2015). The two-speed genomes of filamentous pathogens: Waltz with plants. Curr. Opin. Genet. Dev..

[30] Ma L.-J., Fedorova N.D. (2010). A practical guide to fungal genome projects: Strategy, technology, cost and completion. Mycology.

[31] Dhillon B., Gill N., Hamelin R.C., Goodwin S.B. (2014). The landscape of transposable elements in the finished genome of the fungal wheat pathogen Mycosphaerella graminicola. BMC Genom..

[32] Mat Razali N., Cheah B.H., Nadarajah K. (2019). Transposable Elements Adaptive Role in Genome Plasticity, Pathogenicity and Evolution in Fungal Phytopathogens. Int. J. Mol. Sci..

[33] Nadarajah K., Razali N.M., Cheah B.H., Sahruna N.S., Ismail I., Tathode M., Bankar K. (2017). Draft genome sequence of *Rhizoctonia solani* anastomosis group 1 subgroup 1A strain 1802/KB isolated from rice. Genome Announc..

[34] Blum M., Chang H.-Y., Chuguransky S., Grego T., Kandasaamy S., Mitchell A., Nuka G., Paysan-Lafosse T., Qureshi M., Raj S. (2021). The InterPro protein families and domains database: 20 years on. Nucleic Acids Res..

[35] Muszewska A., Hoffman-Sommer M., Grynberg M. (2011). LTR retrotransposons in fungi. PLoS ONE.

[36] Castanera R., Lopez-Varas L., Borgognone A., LaButti K., Lapidus A., Schmutz J., Grimwood J., Perez G., Pisabarro A.G., Grigoriev I.V. (2016). Transposable elements versus the fungal genome: Impact on whole-genome architecture and transcriptional profiles. PLoS Genet..

[37] Beauregard A., Curcio M.J., Belfort M. (2008). The take and give between retrotransposable elements and their hosts. Annu. Rev. Genet..

[38] Kazazian H.H. (2004). Mobile elements: Drivers of genome evolution. Science.

[39] van Regenmortel M.H., Mahy B.W. (2009). Desk Encyclopedia of Plant and Fungal Virology.

[40] Amyotte S.G., Tan X., Pennerman K., del Mar Jimenez-Gasco M., Klosterman S.J., Ma L.-J., Dobinson K.F., Veronese P. (2012). Transposable elements in phytopathogenic *Verticillium* spp.: Insights into genome evolution and inter-and intra-specific diversification. BMC Genom..

[41] Daboussi M.-J., Capy P. (2003). Transposable elements in filamentous fungi. Annu. Rev. Microbiol..

[42] Fedoroff N.V. (2012). Transposable elements, epigenetics, and genome evolution. Science.

[43] Vukich M., Giordani T., Natali L., Cavallini A. (2009). Copia and Gypsy retrotransposons activity in sunflower (*Helianthus annuus* L.). BMC Plant Biol..

[44] Havecker E.R., Gao X., Voytas D.F. (2004). The diversity of LTR retrotransposons. Genome Biol..

[45] da Silva L.L., Moreno H.L.A., Correia H.L.N., Santana M.F., de Queiroz M.V. (2020). Colletotrichum: Species complexes, lifestyle, and peculiarities of some sources of genetic variability. Appl. Microbiol. Biotechnol..

[46] Ma J., Devos K.M., Bennetzen J.L. (2004). Analyses of LTR-retrotransposon structures reveal recent and rapid genomic DNA loss in rice. Genome Res..

[47] Rep M. (2005). Small proteins of plant-pathogenic fungi secreted during host colonization. FEMS Microbiol. Lett..

[48] Zeng L., Pederson S.M., Kortschak R.D., Adelson D.L. (2018). Transposable elements and gene expression during the evolution of amniotes. Mobile DNA.

[49] Wei M., Wang A., Liu Y., Ma L., Niu X., Zheng A. (2020). Identification of the novel effector RsIA_NP8 in *Rhizoctonia solani* AG1 IA that induces cell death and triggers defense responses in non-host plants. Front. Microbiol..

[50] Li S., Peng X., Wang Y., Hua K., Xing F., Zheng Y., Liu W., Sun W., Wei S. (2019). The effector AGLIP1 in *Rhizoctonia solani* AG1 IA triggers cell death in plants and promotes disease development through inhibiting PAMP-triggered immunity in Arabidopsis thaliana. Front. Microbiol..

[51] Dölfors F., Holmquist L., Dixelius C., Tzelepis G. (2019). A LysM effector protein from the basidiomycete *Rhizoctonia solani* contributes to virulence through suppression of chitin-triggered immunity. Mol. Genet. Genom..

[52] Abdul Malik N.A., Kumar I.S., Nadarajah K. (2020). Elicitor and receptor molecules: Orchestrators of plant defense and immunity. Int. J. Mol. Sci..

[53] Depotter J.R., Zuo W., Hansen M., Zhang B., Xu M., Doehlemann G. (2021). Effectors with different gears: Divergence of Ustilago maydis effector genes is associated with their temporal expression pattern during plant infection. J. Fungi.

[54] King B.C., Waxman K.D., Nenni N.V., Walker L.P., Bergstrom G.C., Gibson D.M. (2011). Arsenal of plant cell wall degrading enzymes reflects host preference among plant pathogenic fungi. Biotechnol. Biofuels.

[55] Couturier M., Navarro D., Olivé C., Chevret D., Haon M., Favel A., Lesage-Meessen L., Henrissat B., Coutinho P.M., Berrin J.-G. (2012). Post-genomic analyses of fungal lignocellulosic biomass degradation reveal the unexpected potential of the plant pathogen *Ustilago maydis*. BMC Genom..

[56] Cantarel B.L., Coutinho P.M., Rancurel C., Bernard T., Lombard V., Henrissat B. (2008). The Carbohydrate-Active EnZymes database (CAZy): An expert resource for glycogenomics. Nucleic Acids Res..

[57] Sista Kameshwar A.K., Qin W. (2018). Comparative study of genome-wide plant biomass-degrading CAZymes in white rot, brown rot and soft rot fungi. Mycology.

[58] Ospina-Giraldo M.D., Griffith J.G., Laird E.W., Mingora C. (2010). The CAZyome of *Phytophthora* spp.: A comprehensive analysis of the gene complement coding for carbohydrate-active enzymes in species of the genus *Phytophthora*. BMC Genom..

[59] Garron M.-L., Cygler M. (2010). Structural and mechanistic classification of uronic acid-containing polysaccharide lyases. Glycobiology.

[60] Sharma A., Tewari R., Rana S.S., Soni R., Soni S.K. (2016). Cellulases: Classification, methods of determination and industrial applications. Appl. Biochem. Biotechnol..

[61] Yennamalli R.M., Rader A.J., Kenny A.J., Wolt J.D., Sen T.Z. (2013). Endoglucanases: Insights into thermostability for biofuel applications. Biotechnol. Biofuels.

[62] Yamamoto N., Wang Y., Lin R., Liang Y., Liu Y., Zhu J., Wang L., Wang S., Liu H., Deng Q. (2019). Integrative transcriptome analysis discloses the molecular basis of a heterogeneous fungal phytopathogen complex, *Rhizoctonia solani* AG-1 subgroups. Sci. Rep..

[63] Davies K., De Lorono I., Foster S., Li D., Johnstone K., Ashby A. (2000). Evidence for a role of cutinase in pathogenicity of Pyrenopeziza brassicae on brassicas. Physiol. Mol. Plant Pathol..

[64] Ramzi A.B., Me M.L.C., Ruslan U.S., Baharum S.N., Muhammad N.A.N. (2019). Insight into plant cell wall degradation and pathogenesis of Ganoderma boninense via comparative genome analysis. PeerJ.

[65] Farrer R.A., Fisher M.C. (2017). Describing genomic and epigenomic traits underpinning emerging fungal pathogens. Adv. Genet..

[66] Sang H., Hulvey J.P., Green R., Xu H., Im J., Chang T., Jung G. (2018). A Xenobiotic Detoxification Pathway through Transcriptional Regulation in Filamentous Fungi. mBio.

[67] Patel D.D., Patel A.K., Parmar N.R., Shah T.M., Patel J.B., Pandya P.R., Joshi C.G. (2014). Microbial and Carbohydrate Active Enzyme profile of buffalo rumen metagenome and their alteration in response to variation in the diet. Gene.

[68] Park Y.-J., Jeong Y.-U., Kong W.-S. (2018). Genome Sequencing and Carbohydrate-Active Enzyme (CAZyme) Repertoire of the White Rot Fungus *Flammulina elastica*. Int. J. Mol. Sci..

[69] Brenelli L.B., Persinoti G.F., Cairo J.P.L.F., Liberato M.V., Gonçalves T.A., Otero I.V., Mainardi P.H., Felby C., Sette L.D., Squina F.M. (2019). Novel redox-active enzymes for ligninolytic applications revealed from multiomics analyses of *Peniophora* sp. CBMAI 1063, a laccase hyper-producer strain. Sci. Rep..

[70] Hu J., Arantes V., Pribowo A., Gourlay K., Saddler J.N. (2014). Substrate factors that influence the synergistic interaction of AA9 and cellulases during the enzymatic hydrolysis of biomass. Energy Environ. Sci..

[71] DeYoung B.J., Innes R.W. (2006). Plant NBS-LRR proteins in pathogen sensing and host defense. Nat. Immunol..

[72] Armenta S., Moreno-Mendieta S., Sánchez-Cuapio Z., Sánchez S., Rodríguez-Sanoja R. (2017). Advances in molecular engineering of carbohydrate-binding modules. Proteins.

[73] Bao J., Chen M., Zhong Z., Tang W., Lin L., Zhang X., Jiang H., Zhang D., Miao C., Tang H. (2017). PacBio sequencing reveals transposable elements as a key contributor to genomic plasticity and virulence variation in *Magnaporthe oryzae*. Mol. Plant.

[74] Omrane S., Audéon C., Ignace A., Duplaix C., Aouini L., Kema G., Walker A.-S., Fillinger S. (2017). Plasticity of the MFS1 promoter leads to multidrug resistance in the wheat pathogen *Zymoseptoria tritici*. MSphere.

[75] de Jonge R., Bolton M.D., Kombrink A., van den Berg G.C., Yadeta K.A., Thomma B.P. (2013). Extensive chromosomal reshuffling drives evolution of virulence in an asexual pathogen. Genome Res..

[76] Kirkland T.N., Muszewska A., Stajich J.E. (2018). Analysis of transposable elements in *Coccidioides* species. J. Fungi.

[77] Chadha S., Sharma M. (2014). Transposable elements as stress adaptive capacitors induce genomic instability in fungal pathogen *Magnaporthe oryzae*. PLoS ONE.

[78] Barber E.A., Liu Z., Smith S.R. (2020). Organic Contaminant Biodegradation by Oxidoreductase Enzymes in Wastewater Treatment. Microorganisms.

[79] Kim Y.H. (2016). Discovery and characterization of new O-methyltransferase from the genome of the lignin-degrading fungus *Phanerochaete chrysosporium* for enhanced lignin degradation. Enzym. Microb. Technol..

[80] Ismail I.A., Able A.J. (2016). Secretome analysis of virulent *Pyrenophora teres* f. teres isolates. Proteomics.

[81] Shim W.-B., Sagaram U.S., Choi Y.-E., So J., Wilkinson H.H., Lee Y.-W. (2006). FSR1 is essential for virulence and female fertility in *Fusarium verticillioides* and *F. graminearum*. Mol. Plant-Microbe Interact..

[82] Soanes D.M., Alam I., Cornell M., Wong H.M., Hedeler C., Paton N.W., Rattray M., Hubbard S.J., Oliver S.G., Talbot N.J. (2008). Comparative genome analysis of filamentous fungi reveals gene family expansions associated with fungal pathogenesis. PLoS ONE.

[83] Cantu D., Vicente A.R., Labavitch J.M., Bennett A.B., Powell A.L. (2008). Strangers in the matrix: Plant cell walls and pathogen susceptibility. Trends Plant Sci..

[84] Park J., Lappe M., Teichmann S.A. (2001). Mapping protein family interactions: Intramolecular and intermolecular protein family interaction repertoires in the PDB and yeast. J. Mol. Biol..

[85] Devi B.S.R., Kim Y.-J., Sathiyamoorthy S., Khorolragchaa A., Gayathri S., Parvin S., Yang D.-U., Selvi S.K., Lee O.R., Lee S. (2011). Classification and characterization of putative cytochrome P450 genes from Panax ginseng CA Meyer. Biochemistry.

[86] Moktali V., Park J., Fedorova-Abrams N.D., Park B., Choi J., Lee Y.-H., Kang S. (2012). Systematic and searchable classification of cytochrome P450 proteins encoded by fungal and oomycete genomes. BMC Genom..

[87] Carbon S., Ireland A., Mungall C.J., Shu S., Marshall B., Lewis S., Hub A., Group W.P.W. (2008). AmiGO: Online access to ontology and annotation data. Bioinformatics.

[88] Daboussi M.J. (1996). Fungal transposable elements: Generators of diversity and genetic tools. J. Genet..

[89] Črešnar B., Petrič Š. (2011). Cytochrome P450 enzymes in the fungal kingdom. Biochim. Biophys. Acta.

[90] Liu D., Hunt M., Tsai I.J. (2018). Inferring synteny between genome assemblies: A systematic evaluation. BMC Bioinform..

[91] Lee D.-Y., Jeon J., Kim K.-T., Cheong K., Song H., Choi G., Koh J., Opiyo S., Zuo S., Madhav M.S. (2019). Comparative genome analyses of four rice-infecting isolates of *Rhizoctonia solani* belonging to anastomosis group 1-intraspecific group IA (RsAG1-IA). Plant Health.

[92] Losada L., Pakala S.B., Fedorova N.D., Joardar V., Shabalina S.A., Hostetler J., Pakala S.M., Zafar N., Thomas E., Rodriguez-Carres M. (2014). Mobile elements and mitochondrial genome expansion in the soil fungus and potato pathogen *Rhizoctonia solani* AG-3. FEMS Microbiol. Lett..

[93] Urban M., Cuzick A., Seager J., Wood V., Rutherford K., Venkatesh S.Y., Silva N.D., Martinez M.C., Helder Pedro A.D.Y., Hassani-Pak K. (2019). PHI-base: The pathogen–host interactions database. Nucleic Acids Res..

[94] Yin Y., Mao X., Yang J., Chen X., Mao F., Xu Y. (2012). dbCAN: A Web Resource For Automated Carbohydrate-active Enzyme Annotation. Nucleic Acids Res..

