# Supplementary material for: Comparative Genomics: Insights on the Pathogenicity and Lifestyle of Rhizoctonia solani
Source: Int J Mol Sci. 2021 Feb 22;22(4):2183. doi: 10.3390/ijms22042183 (PMC7926851; doi:10.3390/ijms22042183)
Supplement: Supplementary file 1 [file ijms-22-02183-s001.zip › Supplementary Table S1.docx]

Supplementary Table S1

Transposable elements (TE) landscape of AG1-IA (UKM) which includes abundance, class and superfamilies assignment.

| **Type of repeat** | **Class** | **count** | **bpMasked** | **%masked** |
| --- | --- | --- | --- | --- |
| Academ-H | DNA (Class 2) | 2 | 153 | 0 |
| CMC-EnSpm | DNA (Class 2) | 39 | 18450 | 0.07 |
| MULE-MuDR | DNA (Class 2) | 128 | 58788 | 0.21 |
| PIF-Harbinger | DNA (Class 2) | 4 | 389 | 0 |
| TcMar | DNA (Class 2) | 1 | 32 | 0 |
| TcMar-Ant1 | DNA (Class 2) | 2 | 83 | 0 |
| TcMar-Fot1 | DNA (Class 2) | 5 | 505 | 0 |
| TcMar-Pogo | DNA (Class 2) | 2 | 173 | 0 |
| TcMar-Sagan | DNA (Class 2) | 6 | 814 | 0 |
| TcMar-Tc1 | DNA (Class 2) | 4 | 1194 | 0 |
| L1-Zorro | LINE (Class 1) | 1 | 51 | 0 |
| R1 | LINE (Class 1) | 73 | 11769 | 0.04 |
| Tad1 | LINE (Class 1) | 26 | 2207 | 0.01 |
| Copia | LTR (Class 1) | 45 | 7245 | 0.03 |
| Gypsy | LTR (Class 1) | 704 | 288449 | 1.03 |
| Helitron | Rolling Circle (Class 2) | 2 | 95 | 0 |
| Unknown | Unknown (Others) | 1614 | 342969 | 1.22 |
| Unspecified | Unspecified (Others) | 2986 | 365509 | 1.3 |
| Low_complexity | Low_complexity (Others) | 693 | 33259 | 0.12 |
| Simple_repeat | Simple_repeat (Others) | 6559 | 266889 | 0.95 |
| rRNA | rRNA (Others) | 5 | 5716 | 0.02 |
|  |  | **12901** | **1404739** | **5** |
