# Supplementary material for: Comparative Genomics: Insights on the Pathogenicity and Lifestyle of Rhizoctonia solani
Source: Int J Mol Sci. 2021 Feb 22;22(4):2183. doi: 10.3390/ijms22042183 (PMC7926851; doi:10.3390/ijms22042183)
Supplement: Supplementary file 1 [file ijms-22-02183-s001.zip › Supplementary Table S2.docx]

Supplementary Table S2

Distribution of TE and superfamilies inserted in proximity with the pathogenicity-associated genes within 5000bp.

|  | **Draft AG1-IA** | **China AG1-IA** | **AG1-IB** | **AG3** | **AG8** |
| --- | --- | --- | --- | --- | --- |
| ***Gypsy*** | 11 | 40 | 16 | 16 | 19 |
| ***Copia*** | 7 | 5 | 3 | 7 | 5 |
| **HelitronORF** | 1 | 3 | 1 | 2 | 9 |
| **Mariner** | - | 6 | 4 | 2 | 8 |
| **MuDR** | - | - | 1 | - | - |
| **CACTA** | 2 | 3 | 10 | 10 | 13 |
| **hAT** | 2 | 2 | 3 | 30 | 12 |
| **DDE1** | - | 2 | 2 | 10 | 6 |
| **Mariner_ant** | - | 1 | - | 11 | 5 |
| **LINE** | 8 | 3 | 6 | 21 | 14 |
| **ISC1316** | - | 2 | - | - | - |
| **TOTAL** | **31** | **67** | **46** | **109** | **91** |
